# Examining the frequency and concomitant dose of geometric verification imaging for patients undergoing proton beam therapy for ocular tumours; an audit of current clinical practice

**DOI:** 10.1259/bjro.20210015

**Published:** 2021-07-05

**Authors:** Kate Shrewsbury-Gee, Daniel Kelly, Mike Kirby

**Affiliations:** 1 The Clatterbridge Cancer Centre, Liverpool, UK; 2 The National Centre for Eye Proton Therapy, The Clatterbridge Cancer Centre, Liverpool, UK; 3 Radiotherapy directorate, School of Health Sciences, Institute of Population Health, University of Liverpool, Liverpool, UK

## Abstract

**Objectives:**

This paper uses clinical audit to determine the extent and dosimetric impact of additional imaging for patients undergoing ocular proton beam therapy who have no clips visible in the collimated beam.

**Methods:**

An audit was conducted on 399 patients treated at The National Centre for Eye Proton Therapy between 3 July 2017 and 14 June 2019. The mean total number of image pairs over the course of treatment for patients with and without clips visible in the collimated beam were compared.

**Results:**

Among 364 evaluable patients, 333 had clips visible in the collimated beam and 31 did not. There was a statistically significant increase of five image pairs required for patients with no clips visible compared with those with clips visible (mean 14.6 *vs* 9.6 image pairs, respectively; *p* = 2.74 × 10^–6^). This equated to an additional 1.5 mGy absorbed dose, representing an increase in secondary cancer induction risk from 0.0004 to 0.0007%.

**Conclusions:**

The small increase in concomitant dose and set-up time for patients with no clips visible in the collimated beam is not clinically significant.

**Advances in knowledge:**

This novel work highlights clinical audit from real on-treatment geometric verification data and frequencies, rather than protocols, for ocular proton beam therapy; something not present in the literature. The simple and straightforward methodology is easily and equally applicable to clinical audits (especially those under Ionising Radiation (Medical Exposure) Regulations) for photon techniques.

## Introduction

Clinical audit and clinical evaluation are necessary parts of the radiotherapy process – for assessing clinical outcomes as well as ensuring that procedures and processes are being followed appropriately.^1–3^ Audit is part of the quality assurance (QA) toolkit for ensuring patient care is in line with best practice standards, that those standards are being maintained and that written evidence can be generated of dose assessment and evaluation for patient pathways.^1,2,4,5^ This is good practice, but also a necessary part of national regulation within the UK.^2,3^ QA, clinical audit and clinical evaluation are all required regulations under the Ionising Radiation (Medical Exposure) Regulations (IR(ME)R) 2017 legislation,^2,3^ for providing evidence of practice for both treatment and concomitant exposures. Justification of both are necessary, including assessing risk from an evaluation of exposures from each imaging modality, combined with audit of real clinical practice.^2,3^

Outside regulation, good practice guidance highlights the need for assessment of concomitant exposures undertaken on-treatment for geometric verification.^4–7^ Audit of practice should be thorough, challenging and frequent, to ensure protocols and standards set are being met and adhere to regulation.^1,4,5^ Audits should include assessment of dose for each concomitant exposure, the risks associated with such exposures, the timing and frequency of imaging (especially repeat imaging) and that dose limits are well documented and being adhered to, in the realities of patient treatments, and not just within the assigned protocols.^4,7^

A number of publications highlight evaluations of concomitant dose for pre-treatment and on-treatment procedures and their associated risks; but these are usually phantom or simulation-based, and primarily evaluate protocols.^8–13^ Ding et al^12^ perhaps give the most comprehensive guidance for quantification, management and strategies for concomitant dose reduction from image-guided radiation therapy (IGRT), emphasising the need for assessing and communicating imaging doses, to enable informed decision-making.

Our simple study here adds to this literature. Although focusing on a different treatment modality (proton beam therapy (PBT)) for which on-treatment geometric verification methods are adequately described elsewhere,^4,14^ here we present a method and example which goes beyond modelled estimations of dose from imaging protocols, into the realities of IGRT strategies and real imaging frequencies. The technique is simple and straightforward in its application, as recommended in national guidance.^1,7^ Although applied here to proton beam therapy for ocular tumours, it is easily applicable for audit of photon techniques, especially those under IRMER within the UK. It adds to a literature base which is distinctly lacking in this regard – very few, if any, publications detail this sort of application for proton or photon beam therapy.

Given the structure of the eye and the consequential close proximity of any tumour to essential ocular structures,^15^ ocular PBT uses submillimetre tolerances for eye positioning.^16^ The geometric verification process for ocular PBT involves radio-opaque tantalum clips, 2.5 mm in diameter, sutured around the tumour,^3^ as fiducial markers. An orthogonal X-ray image pair is acquired on-treatment, and each image matched against the pre-treatment planning images for clip placement. Set-up error is calculated from the difference in alignment of the clips, and patient position (using the treatment chair) is adjusted accordingly. The set-up correction is verified with repeated image acquisition until set-up is acceptable. The maintenance of the accurate eye position is then monitored on a screen linked to a video camera. The eye features are outlined on the screen immediately prior to the acquisition of each X-ray image pair and closely monitored once the position is verified.

**Figure 1.**
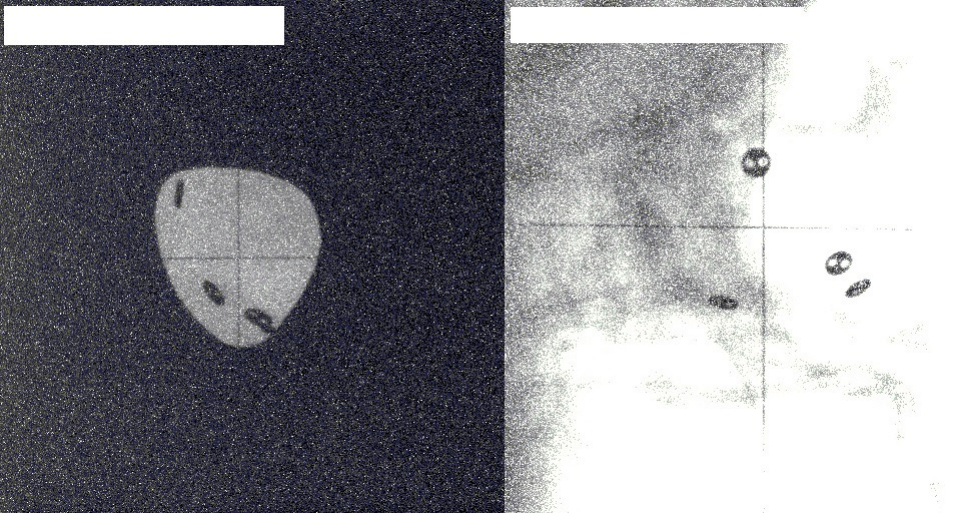
An image pair for a patient with clips visible in the collimated beam. The left image shows the anteroposterior view, the right image shows the lateral view. The collimator is on Images acquired at The National Centre for Eye Proton Therapy, with permission.

**Figure 2.**
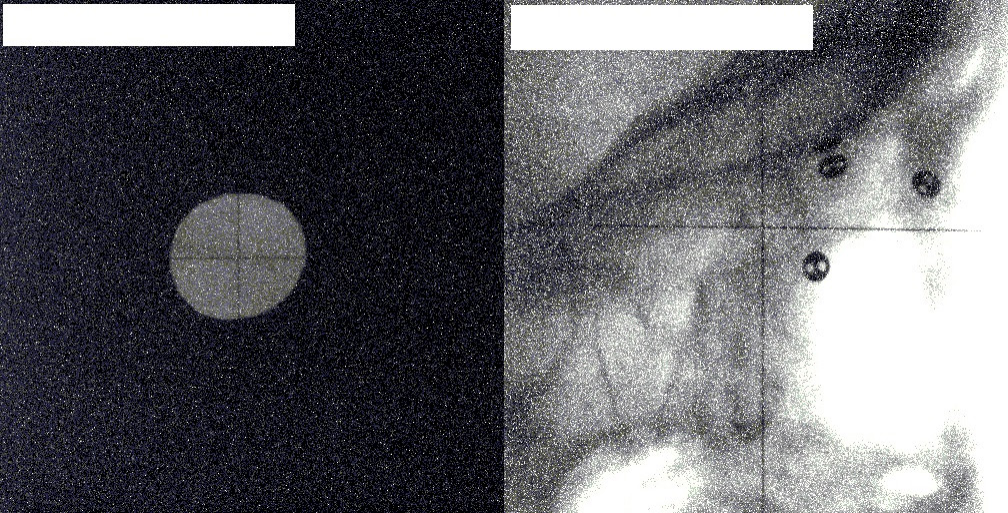
An image pair for a patient with no clips visible in the collimated beam. The left image shows the anteroposterior view, the right image shows the lateral view. The collimator is on, resulting in no clips being visible on the anteroposterior image Images acquired at The National Centre for Eye Proton Therapy, with permission.

**Figure 3.**
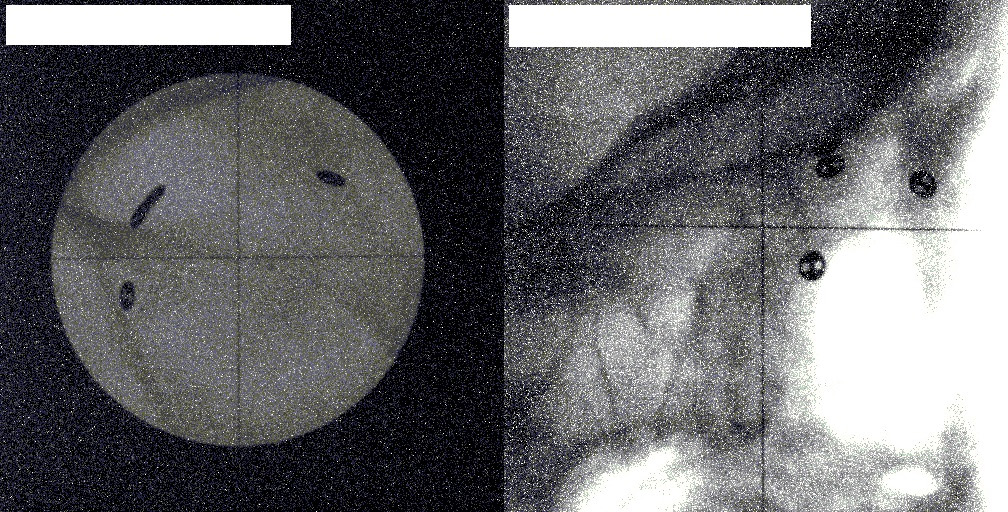
An image pair for a patient with no clips visible in the collimated beam (the same patient as in Figure 2). The left image shows the anteroposterior view, the right image shows the lateral view. The collimator is off so that the clips are visible on the anteroposterior image Images acquired at The National Centre for Eye Proton Therapy, with permission.

For most patients, clips are visible through the collimator on both anteroposterior (AP) and lateral set-up images (Figure 1). However, when clips cannot be sutured close to the tumour borders (particularly difficult for posterior or diffuse tumours),^17^ they are only visible for the lateral image due to the shape of the collimator (Figure 2). Moves in the left–right direction cannot be seen on the lateral images, so the first image pair must be acquired without the collimator (Figure 3). Positioning the collimator may cause some patient movement, so another image pair must be acquired to verify patient position once the collimator has been placed. This will verify the correct position in the Y and Z co-ordinates, with the outlined eye features on the screen acting as a proxy for the X co-ordinate. This process results in patients with no clips visible in the collimated beam having a minimum of two image pairs per treatment as opposed to one for patients with clips visible in the collimated beam. If a left–right move is suspected (due to deviation from the outlined features) whilst the collimator is on, it must be removed for the acquisition of another image pair, and consequently an additional image pair must be acquired to verify position once it has been placed again. This means that patients with no clips visible in the collimated beam require additional imaging, the extent of which and dosimetric impact are the subject of this study.

The dose from these set-up images contribute to the overall concomitant dose for the patient – that which is extra to the prescribed treatment dose.^4,5^ These exposures are essential in ensuring the accurate delivery of treatment, resulting in an overall benefit to the patient,^4^ but, as mentioned, should be audited, assessed and justified under IR(ME)R.^2–4^ Practitioners have a responsibility to keep the dose to the patient as low as reasonably practicable.^2–4^ Although the dose from the X-ray images is likely small in comparison to the prescribed treatment dose, any amount of ionising radiation can lead to adverse clinical effects for patients.^4^ For the patients with ocular tumours under discussion here, the clinical consequences may include the induction of secondary cancer or the formation of cataracts.

This study aimed to determine the extent of the increase in image pairs for patients with no clips visible in the collimated beam, and to evaluate the clinical significance and risk of this added concomitant dose for real imaging frequencies.

## Methods

### Cohort

Data were collected retrospectively from 399 patients who received treatment for ocular tumours at The National Centre for Eye Proton Therapy between 3 July 2017 and 14 June 2019. Patients having treatment to the iris were excluded as these tumours are visible to the naked eye and can be set up using the field light, negating the need for clips. Those patients having clips but without a full set of data recorded were excluded.

### Set-up and treatment information

The dose per orthogonal X-ray image pair had been previously measured as 0.3 mGy. Standard imaging settings were 80 kV/25 mAs for the axial X-ray and 70 kV/5 mAs for the lateral. The field size was approximately 6 × 6 cm.

The majority of patients received the standard dose of 52 Gy (those being treated for benign conditions received 18 Gy) in 4 consecutive daily fractions.

### Data extraction

The data collected for each patient from their treatment records were:

- Whether or not the patient had clips visible in the collimated field.
- The number of image pairs required for position verification. The number of set-up image pairs for each of the four treatments was recorded.

An AP set-up image showing less than half a clip visible in the collimated beam, was defined as an image with ‘no clips visible’, indicating the need to remove the collimator and acquire another image. Uncertainties regarding clip visibility were additionally reviewed by a medical physicist experienced in ocular PBT and clinical image review. Data were gathered in a reverse chronological order, starting with those most recently treated in the cohort.

### Ethics

For this non-experimental, retrospective study, no ethical approval or informed consent was deemed necessary. Both the treatment delivery and recording of data were conducted as part of routine clinical practice, independent of the study. All data collected were anonymised, no patient identifiers were recorded outside of the secure trust systems and network, and no patient can be identified by any of the data or analysis contained within this work.

### Data analysis

The data, collected by hand, was entered into Excel to facilitate analysis. Patients were divided into two groups, according to whether or not they had clips visible in the collimated beam.

The upper and lower bounds of the data were calculated and values outside of these were excluded as outliers. This was done for each group of patients separately. 36 outliers were identified, resulting in 363 patients being included in the final analysis. The total number of set-up image pairs for each patient was calculated, and the mean total number of set-up images for the full course of treatment for both groups were calculated and compared.

### Statistical analysis

An independent, one-tailed *t*-test assuming unequal variances was used to determine any statistically significant difference between the two groups in the number of image pairs recorded. Statistical significance was defined as results where *p* < 0.05.

## Results

Among the 363 patients analysed, 332 had clips visible in the collimated beam and 31 had no clips visible in the collimated beam. The mean total number of image pairs over the course of treatment recorded in these two groups were 9.6 (range 5–29) and 14.6 (range 7–26), respectively. This increase was statistically significant (*p* = 2.74 x 10^−6^).

## Discussion

These findings indicate that patients with no clips visible in the collimated beam require, on average, five additional image pairs over the course of treatment when compared with patients with clips visible in the collimated beam. This equates to exposure to an additional 1.5 mGy.

### Radiation-induced carcinogenesis

In comparison to the high doses of radiation generally associated with increased risk of secondary cancer, 0.3 mGy per image pair for ocular PBT may seem negligible. However, carcinogenesis is a stochastic effect, meaning the risk increases as the dose increases.^18^ Therefore, *any* additional dose will increase the risk of secondary cancer induction. Additionally, there are certain carcinogenic effects that take place at low doses,^19^ and evidence shows an increased incidence of cancer after low doses of radiation (less than 2 Sv).^20^

To gain an understanding of the significance of the extra imaging dose in terms of carcinogenesis, the risk of secondary cancer was calculated (Table 1) using the methodology of Waddington and McKenzie.^21^ The average concomitant dose was calculated by multiplying the number of image pairs by 0.3 mGy (the dose from each image pair). The effective dose was calculated by multiplying the average concomitant dose by a radiation weighting factor (one for photons^22^) and then multiplying by a tissue weighting factor. Here, the probability is calculated for skin, bone surface, and brain (tissues likely to be irradiated during ocular imaging). These tissues have an individual weighting factor of 0.01 each,^23^ resulting in an overall tissue weighting factor of 0.03. The effective dose was multiplied by 0.005 (the probability that 1 mSv will induce a fatal cancer in an average person^21^) to give the probability of secondary cancer induction.

**Table 1.** Chance of secondary cancer induction due to imaging dose

	Clips visible in the collimated beam	No clips visible in the collimated beam	Maximum number of image pairs allowed by protocol	Maximum number of image pairs allowed by protocol (with additional five authorised and supervised by MPE)
Average concomitant dose	2.9 mGy	4.4 mGy	12 mGy	18 mGy
Effective dose	0.087 mSv	0.132 mSv	0.36 mSv	0.54 mSv
Probability of secondary cancer induction	0.0004 %	0.0007 %	0.0018 %	0.0027 %

MPE, medical physics expert.

Table 1 shows that on average, the increased risk of secondary cancer due to increased concomitant dose is 0.0003%, rising from 0.0004% in patients with clips visible in the collimated beam to 0.0007% in those without clips visible. This increase is considerably lower than Waddington and McKenzie’s suggested action level of 0.5%.^21^ For comparison, the average human living in the UK receives 2.7 mSv per year background radiation^24^: more than 20 times the concomitant imaging dose received by a patient with no clips visible in the collimated beam.

The latter two columns of Table 1 show the risk of secondary cancer from the maximum number of image pairs allowed by the centre’s protocol. No patient over the time period audited went above the number of image pairs authorised by the protocol. The maximum number of image pairs required over the course of treatment for any patient over this time period was 29: just below half of the maximum number of images authorised by the protocol (with the presence of a medical physics expert). This verifies that the concomitant dose is always within acceptable limits as defined by the centre and indeed there is room for a possible tightening of the tolerance.

### Radiation-induced cataractogenesis

Aside from the risk of carcinogenesis, radiation dose to the eye can result in the formation of cataracts.^25^ Doses as low as 0.5 Gy can promote increased and earlier cataract development,^26^ and research suggests that cataractogenesis may be a stochastic effect rather than deterministic as previously thought.^25^ However, it is likely that cataract formation is determined by whether or not the treatment beam passes through the lens, and that the relatively low concomitant imaging dose will not contribute significantly to this.

### Limitations

This study was retrospective, which can never definitely establish a causal relationship. This study has not considered other factors (such as anxiety) that may lead to an increase in image pairs. While the removal of outliers with unusually high or low increases in imaging frequency may help in part to account for this, the potential impact on the results of including patients with smaller deviations from the mean cannot be discounted.

## Conclusion

This audit has found that for this ocular PBT technique, patients with no clips visible in the collimated beam require more set-up images than patients with clips visible in the collimated beam. Although secondary cancers are a known and unavoidable consequence of radiotherapy treatment, the additional concomitant dose received by patients with no clips visible in the collimated beam is not deemed clinically significant and is well within the centre’s protocol. In addition, this paper has highlighted that the methodology used is straightforward, being able to be used to audit real image frequencies and the consequent risk from a real IGRT strategy. No similar studies have been reported within the literature, to the authors’ knowledge, using this highly accessible, simple method; one which could be easily used for other external beam radiotherapy techniques for photon therapy as well as PBT, for general audit purposes and audits under IRMER. For example, the method could be applied to image numbers recorded through the oncology management system for different clinical sites (perhaps in an automated way by interrogating the database) and risk assessed in a similar manner to that used here. Although the results verify that the concomitant doses from this on-treatment geometric verification method are within protocol limits, changes in practice (image number limits) could be considered. This could trigger audit and investigation earlier, having the benefit of uncovering potentially hidden issues within the radiotherapy process that might be the cause of more frequent on-treatment imaging.
